# Prostate shapes on pre-treatment MRI between prostate cancer patients who do and do not undergo biochemical recurrence are different: Preliminary Findings

**DOI:** 10.1038/s41598-017-13443-8

**Published:** 2017-11-20

**Authors:** Soumya Ghose, Rakesh Shiradkar, Mirabela Rusu, Jhimli Mitra, Rajat Thawani, Michael Feldman, Amar C. Gupta, Andrei S. Purysko, Lee Ponsky, Anant Madabhushi

**Affiliations:** 1 Case Western Reserve University, Department of Biomedical Engineering, Cleveland, OH 44106 USA; 2 Section of Abdominal Imaging, Imaging Institute, Cleveland Clinic, Cleveland, OH 44195 USA; 3 Urology Institute, University Hospitals Cleveland Medical Center, Case Western Reserve University School of Medicine, Cleveland, USA; 4 Case Western Reserve University, Department of Biomedical Engineering, Niskayuna, NY 12309 USA; 5 University of Pennsylvania, Perelman School of Medicine, Philadelphia, PA 19104 USA; 6 Present Address: GE Global Research, Niskayuna, NY 12309 USA

## Abstract

Early identification of PCa patients at risk for biochemical recurrence (BCR) post-therapy will potentially complement definitive therapy with either neo- or adjuvant therapy to improve prognosis. BCR post definitive therapy is often associated with disease progression that might cause a bulge in the prostate gland. In this work we explored if an atlas-based comparison approach reveals shape differences in the prostate capsule as observed on pre-treatment T2-weighted MRI between prostate cancer patients who do (*BCR* ^+^) and do not (*BCR* ^−^) have BCR following definitive therapy. A single center IRB approved study included 874 patients. Complete image datasets, clinically localized PCa, availability of Gleason score, data available for post-treatment PSA and follow-up for at least 3 years in patients without BCR were the inclusion criteria to select 77 patients out of the 874 patients. Further controlling for Gleason score, stage, age and to maintain equal number of cases for the *BCR* ^+^ and *BCR* ^−^ categories, the total number of cases was reduced to 50. Manually segmented prostate capsules were aligned to a *BCR* ^−^ template for statistical comparison between the *BCR* ^+^ and *BCR* ^−^ groups. Statistically significant shape difference between the two groups was observed towards the lateral and the posterior sides of prostate.

## Introduction

About 180,890 new cases of prostate cancer will be diagnosed in USA in 2016^1^. Over 50% of these patients will be treated with either radical prostactomy or radiation therapy or both^2^. Despite advancement in surgical procedures and radiation therapy, there may be an treatment failure in estimated 30–35% of the treated prostate cancer patients within 10 years time^3^. An elevated prostate specific antigen (PSA), 0.2 ng/ml for surgery or 2 ng/ml for radiation therapy above the nadir is indicative of treatment failure or biochemical recurrence (BCR). BCR is often associated with the presence of more aggressive prostate cancer and early detection of biochemical recurrence in men with prostate cancer undergoing definitive therapy may help identify patients who would benefit from adjuvant or neo-adjuvant therapies.

Pre-treatment nomograms such as the one by Kattan *et al*.^4^ have employed clinical variables such as pre-treatment PSA, biopsy, Gleason score, clinical stage and radiation dose to predict risk of BCR within five-years of definitive therapy. Park *et al*.^5^ found statistically significantly lower mean Apparent Diffusion Coefficient (ADC) values within the tumor region for patients with BCR. In one of our recent study^6^ statistically significant differences were observed in prostate shape on T2w images between patients with and without prostate cancer.

Extracapsular extent is frequently associated with the risk of developing BCR^7^. Extracapsular extent often results in an irregular buldge in the prostate and may cause focal capsular retraction^8^. Thus, prostate cancer may affect the shape of the prostate capsule as it locally and internally pervades the gland. In^6^, Rusu *et al*. showed via an atlas based approach that the shape of the prostate capsule and the transition zone as observed on a T2w MRI were significantly different between men with and without prostate cancer. Building on the findings of^6^, in this work we sought to explore whether an atlas based comparison approach could help identify shape differences in the prostate capsule as observed on pre-treatment T2 weighted MRI between prostate cancer patients who do (*BCR* ^+^) and do not (*BCR* ^−^) have BCR following definitive therapy. Unlike the work showcased in Rusu *et al*. which revealed that shape differences exist between diseased and a normal prostate, the objective of this study was to spatially localize surface of interest that significantly differs between (*BCR* ^+^) and (*BCR* ^−^) patients, while controlling for ECap in the process. The difference in the surface of interest information could then potentially be used as a bio-marker to differentiate (*BCR* ^+^) and (*BCR* ^−^) cohorts in an independent validation set in the future. Another crucial difference between our approach and that of Rusu *et al*. is that our framework is completely automatic and a robust GLM based T-test method is used to interrogate shape differences between (*BCR* ^+^) and (*BCR* ^−^) cases.

*BCR* ^+^ and *BCR* ^−^ patients were identified and selected from a retrospective group of prostate cancer patients who had previously undergone definitive therapy and for whom the outcomes had been recorded, all under the auspices of an IRB approved study. An experiences genitourinary radiologist in prostate MRI manually contoured the prostate gland in T2w images. All prostate capsules from across the different patients were aligned for statistical comparison. The *BCR* ^+^ and *BCR* ^−^ cohorts were compared using a Generalized Linear Model (GLM) based t-test corrected for multiple comparison to identify the regions of the prostate capsule that significantly differs between the two cohorts. Shape differences between the two populations was also studied while controlling for extra-capsular spread. The entire framework for the study is presented in Fig. 2. All notations used in this paper are summarized in Table 1.

**Table 1:** Notations used in the paper.

Acronym	Definition
BCR	Biochemical recurrence
ECap	Extra-capsular extent
*BCR* ^+^	Patients with biochemical recurrence
*BCR* ^+^	Patients without any biochemical recurrence
*BCR* ^+^_*ECap*−_	Patients with biochemical recurrence and without extracapsular extent
*BCR* ^−^_*ECap*−_	Patients without biochemical recurrence and without extracapsular extent
*BCR* ^+^_*ECap*+_	Patients with biochemical recurrence and with extracapsular extent
*BCR* ^−^_*ECap*+_	Patients without biochemical recurrence and with extracapsular extent
*A*	A *BCR* ^−^ template outside the patient cohort to which all patients were registered before atlas creation and statistical comparison

## Materials and Method

### Materials

**Figure 1:**
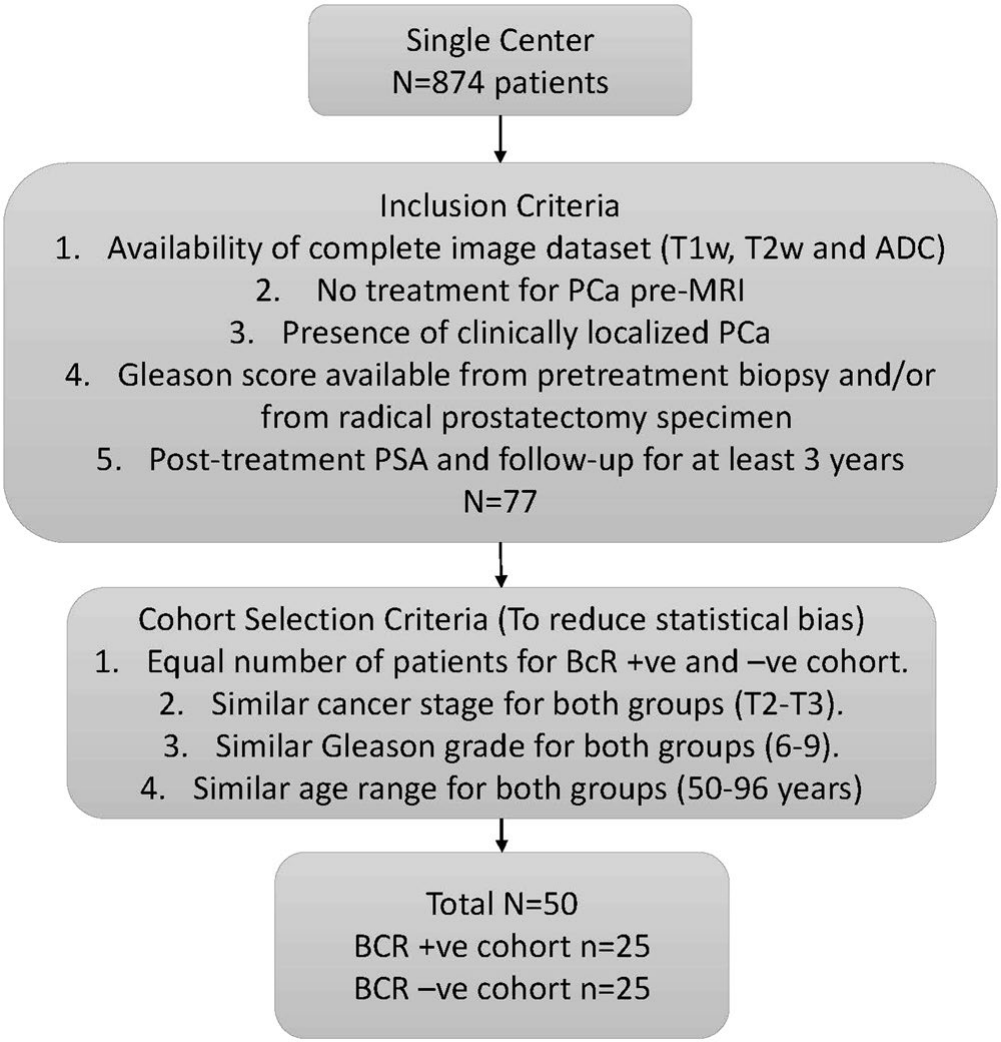
*BCR* ^+^ and *BCR* ^−^ cohort selection criteria.

This study was compliant with the Health Insurance Portability and Accountability Act and was approved by the local Institutional Review Board (IRB) of Cleveland Clinic Foundation, Cleveland, Ohio. Informed consent was waived by the IRB because of the retrospective nature of the study. A search of a prostate MRI registry from a single center included 874 patients referred for a prostate MRI between 2008 and 2014. Availability of complete image datasets (T1w, T2w and ADC map); no treatment for PCa before MRI; presence of clinically localized PCa; availability of Gleason score from pretreatment biopsy and/or from radical prostatectomy specimens; and post-treatment outcome data including post-treatment PSA and a minimum of 3 years of follow-up were used as inclusion criteria. Of the 874 patients, 77 cases met these criteria. *BCR* ^+^ and *BCR* ^−^ cases were selected from these 77 patients. To reduce statistical bias, the following rules were followed for cohort selection: a) equal number of patients in *BCR* ^+^ and *BCR* ^−^ groups; b) similar Gleason scores of 6 to 9 in both groups; and c) similar tumor stages of T2 to T3 in both groups. These added constraints further reduced the final number of patients to 50. Of these 50 patients, 25 were *BCR* ^+^ and 25 were *BCR* ^−^. The *BCR* ^+^ patients ranged from 50 to 96 years, had a Gleason score ranging from 6 to 9, and had T2- to T3-staged disease with a mean recurrence time of 18.5 months. The *BCR* ^−^ patients ranged from 51 to 86 years, had a Gleason score ranging from 6 to 9, and had T2- to T3-staged disease with a mean follow-up time of 4.2 years. The inclusion and exclusion criteria are presented in Fig. 1.

Patient distribution of the *BCR* ^+^ and the *BCR* ^−^ cohorts according to PSA and Gleason scores are presented in Table 2

**Table 2:** Patient distribution of the *BCR* ^+^ and the *BCR* ^−^ cohorts according to PSA and Gleason score. Mean and standard deviation of PSA and Gleason scores of each cohort is presented.

Cohorts	PSA	Gleason
*BCR* ^+^	24.29 ± 45.12	8.63 ± 0.72
*BCR* ^−^	7.07 ± 8.17	7.09 ± 0.76

All patients underwent pretreatment MRI on a 3.0T MR scanner equipped with an endorectal coil and pelvic phased-array coil. T2w turbo spin-echo images were acquired with TR/TE of 4754/115 ms, image resolution of 0.3 mm, and inter-slice thickness of 3 mm. An experienced genitourinary radiologist with more than 7 years of experience in reading prostate MRI reviewed the T1w and T2w images before manually contouring the prostate gland on and the transition zone on T2w images. A second expert’s annotation of the same dataset were acquired to quantify the inter-observer variability of annotation of the prostate capsule. The second expert was a MD with two years of experience in interpreting and reading prostate MRI.

### Method

#### Experimental Design

All prostate MRIs were first aligned/registered to a common canonical frame for statistical comparison. Alignment of all T2w prostate MRI were done in two stages. In the first stage all prostate MRI for a given subpopulation (*BCR* ^+^ or *BCR* ^−^) were aligned to the representative template of that group to create a representative atlas of each of the *BCR* ^+^ and *BCR* ^−^ groups. In the second stage the *BCR* ^+^ and *BCR*−^-^ atlases were registered to a common space for statistical analysis and comparisons. Different atlases were also created for statistical comparison within the *BCR* ^+^ and *BCR* ^−^ groups to control for ECap while exploring the differences in shape between the *BCR* ^+^ and *BCR* ^−^ groups. Thus we also compared shape differences between patients (1) with BCR and with ECap (*BCR* ^+^_*ECap*+_) vs patients without BCR and with ECap (*BCR* ^−^_*ECap*+_), (2) with BCR and without ECap (*BCR* ^+^_*ECap*−_) and without BCR and without ECap (*BCR* ^−^_*ECap*−_). Table 1 contains details of the specific comparisons performed.

To investigate the impact of template selection on our statistical analysis, in an independent experiment, both *BCR* ^+^ and *BCR* ^−^ cohorts were registered to a *BCR* ^−^ patient template that was not included in the 25 patient *BCR* ^−^ cohort. The entire analysis as above i.e., shape comparison between patients (1) *BCR* ^+^ and *BCR* ^−^ groups (2) with BCR and with ECap (*BCR* ^+^_*ECap*+_) vs patients without BCR and with ECap (*BCR* ^−^_*ECap*+_), (3) with BCR and without ECap (*BCR* ^+^_*ECap*−_) and without BCR and without ECap (*BCR* ^−^_*ECap*−_) was repeated.

The atlas and shape based comparison framework can thus be divided into two parts, atlas construction and the statistical comparison of the atlases. The two modules are described below.

#### Atlas Construction

In the initial pre-processing stage, a N4 bias field correction^9^ algorithm was applied to all T2w prostate MRI. To create subpopulation atlases of each of the groups, two approaches were adopted; (1) all prostates inside a given subpopulation (i.e. *BCR* ^+^ or *BCR* ^−^) were registered to the representative template. The prostate with median volume for each of the group was selected as the representative template. A manually segmented mask of the prostate was provided inside the registration framework for registration initialization and was used to provide anatomical constraints and improve registration accuracy. Thus mask was used as an additional channel inside the registration framework. Registration of the prostate to the representative template was performed in two stages, an initial affine registration was performed followed by a non-rigid registration.

A block matching strategy described in^10^ was adopted to determine the transformation parameters for the affine registration. Similarity between a block from the moving image to all blocks of similar dimension in the fixed or the reference image was computed. The best corresponding block defined the displacement vector for the affine transformation. Normalized cross correlation based similarity was used to determine the block correspondences. The affine registration of the moving image to the reference image was followed with a B-spline^11^ based non-rigid registration. This strategy enabled the construction of the different atlases listed in Table 1.

To evaluate whether observed differences were on account of the template selection for atlas creation, we registered all *BCR* ^+^ and *BCR* ^−^ patients to a single template *A*; this template was selected from outside the *BCR* ^+^ and *BCR* ^−^ cases considered in this study. Affine registration followed by a deformable registration (as before) was used to register all patients to *A*. Individual subpopulation atlases of *BCR* ^+^ and *BCR* ^−^ cohorts were created after registration for statistical comparison.

The registration error for atlas creation were quantified with Dice similarity coefficient (DSC) (a overlap measure between the reference template mask and the registered masks), mean absolute surface distance (mean Euclidean surface distance between the reference template mask and the registered masks) and difference in center of gravity (COG) (mean Euclidean distance of COG between the reference template mask and the registered masks).

#### Statistical Comparison

All registered prostate capsules of both the *BCR* ^+^ and *BCR* ^−^ groups were isotropically scaled with 0.3 mm3 resolution and transformed into a signed distance function in the registered space. As opposed to the binary representation of a mask where each voxel within the prostate capsule has a value of 1 and a value of 0 outside the capsule, the value assigned to each voxel was determined based off the distance of a given voxel from the capsule boundary. Consequently, the signed distance function yields positive values for voxels inside the prostate capsule, while the value of the voxel decreases as it approaches the boundary where the signed distance function is zero, becomes negative outside the prostate capsule, and continue to decrease depending on the distance of the voxel from the prostate capsule.

The statistical comparisons of *BCR* ^+^ and *BCR* ^−^ cohorts was done using the corresponding signed distance representations of the voxels^12^ of each prostate capsule registered to the corresponding representative template via a non-parametric Generalized Linear Model (GLM) based t-test^13^ with 5000 random permutation testing and corrected for multiple comparison. In this GLM-based method, permutation testing was used for inference on statistic maps when the null distribution was not known. The method provides a test statistic image and sets of p-values. GLM based on the contrast was automatically partitioned into tested effects and nuisance (confounding) effects. The data were first fit to the nuisance effects alone and nuisance-only residuals were identified. These residuals were permuted, and then the estimated nuisance signal was added back on, creating an (approximate) realization of data under the null hypothesis. This realization was fit to the full model and the desired test statistic was computed as usual. This process was repeated to build a distribution of test statistics equivalent under the null hypothesis specified by the contrast(s). The p-values obtained from the model were corrected for multiple comparisons and the clustering forming threshold was determined automatically using the threshold free cluster enhancement (TFCE) method^13^.

**Figure 2:**
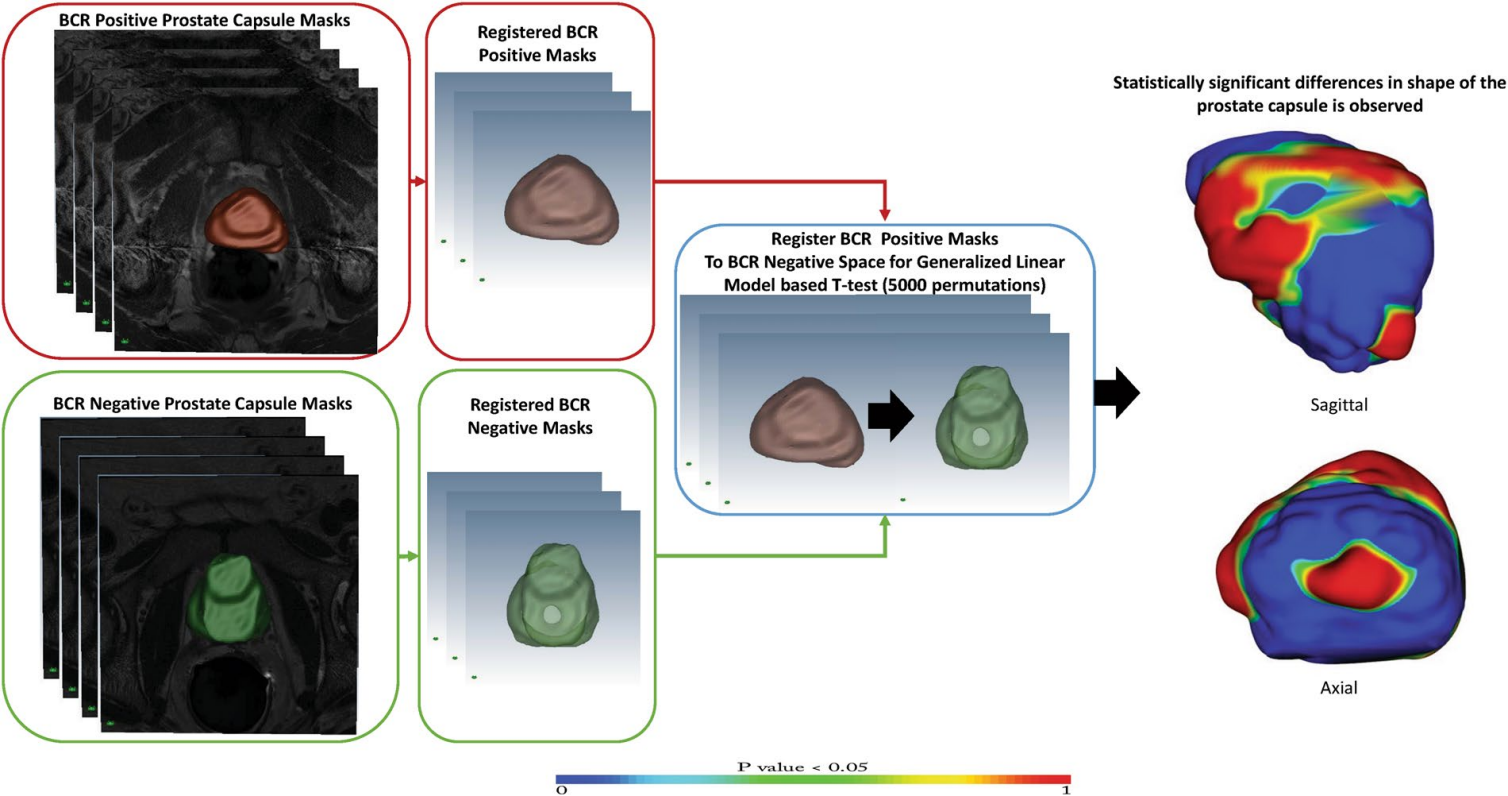
Two separate atlases are created for *BCR* ^+^ and *BCR* ^−^ cases are created. The atlases are co-registered to identify regions that are significantly different between the two atlases. GLM based T-test with 5000 permutation testing and corrected for multiple comparison reveals significant shape differences as observed in the sagittal and the axial view on the right.

Permutation inference is grounded on exchangeability under the null hypothesis, that data can be permuted (exchanged) without affecting its joint distribution. However, if a nuisance effect is present in the model, the data cannot be considered exchangeable even under the null hypothesis^13^. In our model, however, number of patients, cancer stage, Gleason grade and age range was kept similar to minimize the impacts of the nuisance variables. In absence of nuisance variables, our model reduced to a linear model with complete exchangeability. Hence a voxel was considered as belonging to a region exhibiting statistically significant differences between shapes for *BCR* ^+^ and *BCR* ^−^ patients if the p-value estimated by this testing was less than 0.05. The entire framework for prostate capsule shape analysis is presented in Fig. 2

The statistical comparison framework was repeated for subpopulation atlases created after registration of both the cohorts to a *BCR* ^−^ template to investigate the impact of template selection.

## Results

In Table 3 the registration accuracy in terms of DSC, MASD and COG is observed to be high across all the atlases (*BCR* ^−^, *BCR* ^+^, *BCR* ^−^_*ECap*−_, *BCR* ^+^_*ECap*−_, *BCR* ^−^_*ECap*+_, *BCR* ^+^_*ECap*+_). The minimum average DSC value was 0.95 for all the atlases created that may suggest a high overlap ratio and a high registration accuracy. The maximum mean MASD for all the atlases was 0.62 mm with a maximum mean COG difference of 1.03 mm. This may suggest that the preceding registration step is sufficiently accurate to proceed with subsequent statistical analysis and comparative studies across the different prostate capsule shapes. To further quantify the registration error, manually segmented masks of the transition zone was registered to the template. The transition zone mask was not used for registration initialization and was transformed using the same transformation as the prostate capsule. After registration to the template the errors in terms of MASD and COG differences were computed. These differences are reported in Tables 3 and 4.

**Table 3:** Quantitative comparison of registration error for all atlases when registered to *BCR* ^−^ and *BCR* ^+^ templates.

Sub-group Atlases	*DSC* ^WG^	*MASD* ^WG^	*COG* ^WG^	*MASD* ^TZ^	*COG* ^TZ^
*BCR* ^−^	0.97 ± 0.01	0.40 ± 0.14	0.51 ± 0.36	2.05 ± 0.06	0.57 ± 0.09
*BCR* ^+^	0.98 ± 0.006	0.30 ± 0.11	0.11 ± 0.18	1.87 ± 0.12	0.55 ± 0.12
*BCR* ^−^_*ECap*−_	0.95 ± 0.02	0.62 ± 0.31	1.03 ± 1.11	2.06 ± 0.03	0.62 ± 0.10
*BCR* ^+^_*ECap*−_	0.98 ± 0.006	0.28 ± 0.09	0.12 ± 0.23	1.67 ± 0.52	0.44 ± 0.08
*BCR* ^−^_*ECap*+_	0.95 ± 0.02	0.62 ± 0.31	1.03 ± 1.11	2.07 ± 0.44	0.64 ± 0.15
*BCR* ^+^_*ECap*+_	0.98 ± 0.005	0.31 ± 0.20	0.15 ± 0.21	1.86 ± 0.36	0.56 ± 0.12

MASD and COG are reported in mm. A high degree of registration accuracy for atlas creation is observed. WG represents the entire capsule and TZ represents the transition zone.

**Table 4:** Quantitative comparison of registration error for all atlases registered to a *BCR* ^−^ template outside the cohort.

Sub-group Atlases	*DSC* ^WG^	*MASD* ^WG^	*COG* ^WG^	*MASD* ^TZ^	*COG* ^TZ^
*BCR* ^−^	0.96 ± 0.01	0.44 ± 0.17	1.82 ± 0.18	2.38 ± 0.38	0.66 ± 0.15
*BCR* ^+^	0.97 ± 0.01	0.45 ± 0.17	1.90 ± 0.17	2.52 ± 0.55	0.60 ± 0.14
*BCR* ^−^_*ECap*−_	0.97 ± 0.01	0.47 ± 0.23	1.80 ± 0.18	2.29 ± 0.33	0.66 ± 0.14
*BCR* ^+^_*ECap*−_	0.95 ± 0.01	0.49 ± 0.05	1.89 ± 0.10	2.21 ± 0.44	0.63 ± 0.13
*BCR* ^−^_*ECap*+_	0.96 ± 0.01	0.42 ± 0.13	1.85 ± 0.15	2.37 ± 0.44	0.64 ± 0.15
*BCR* ^+^_*ECap*+_	0.97 ± 0.01	0.40 ± 0.13	1.92 ± 0.12	2.53 ± 0.40	0.57 ± 0.16

MASD and COG are reported in mm. Again a high degree of registration accuracy for atlas creation is observed.

In Table 4 the registration accuracy in terms of DSC, MASD and COG is presented when both *BCR* ^−^ and *BCR* ^+^ cohorts are registered to *A*, where *A* was a prostate MRI study from outside the *BCR* ^−^ and *BCR* ^+^ cases considered in this study. A high DSC and MASD values may suggest that the preceding registration step is sufficiently accurate to proceed with subsequent statistical analysis and comparative studies when registered to *A*.

### Shape differences

**Figure 3:**
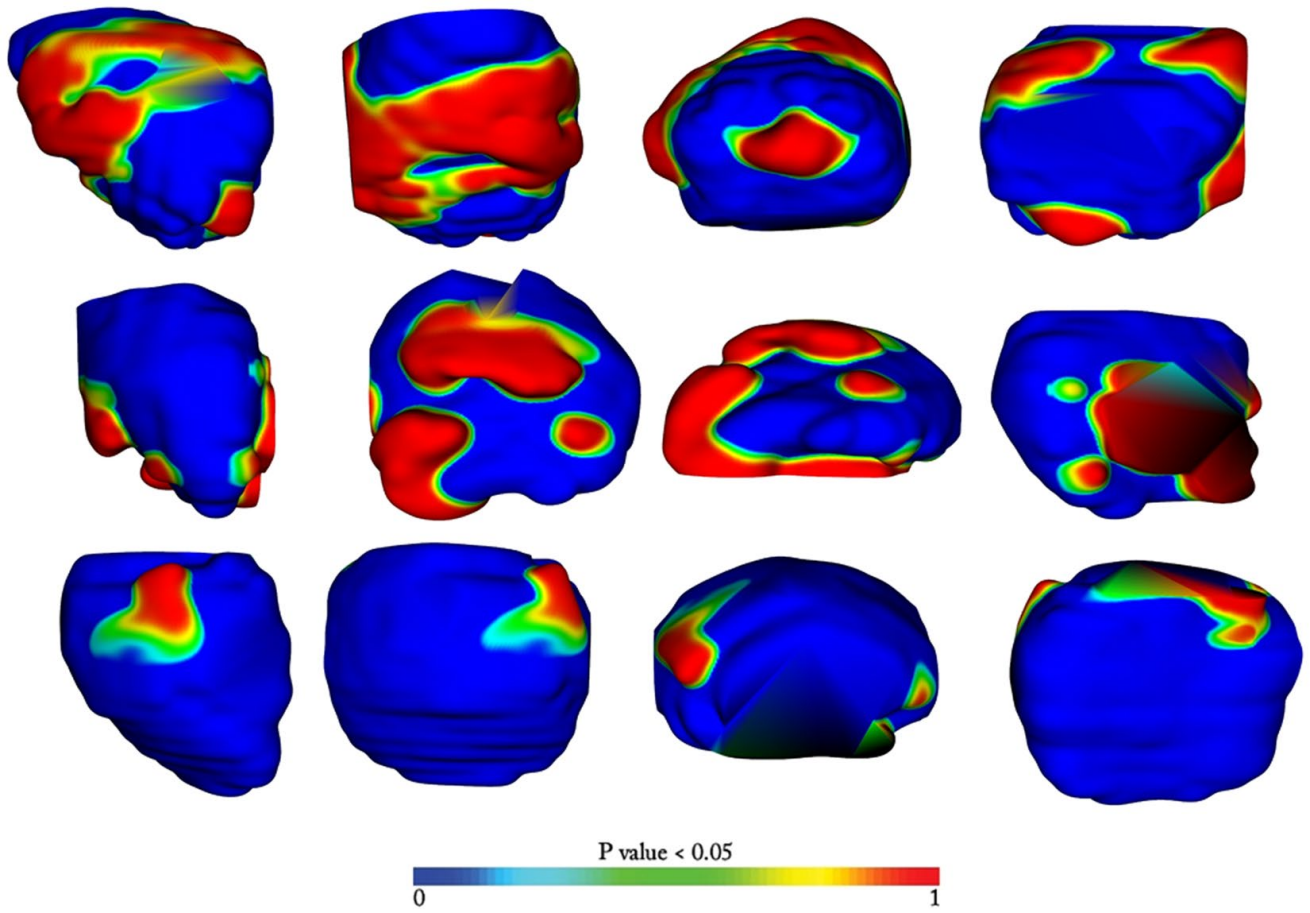
Shape differences of the prostate capsule in the *BCR* ^−^ template space. All p-values are mapped to atlases and thresholded over 0.95. Blue value signifies low difference and red value signifies a bigger difference. The color bar is provided for reference. In row 1 we have shape differences when all *BCR* ^+^ and *BCR* ^−^ cases are compared. In row 2 we have shape difference between *BCR* ^−^ and *BCR* ^−^ with extra capsular extensions (*BCR* ^−^_*ECap*+_ vs *BCR* ^+^_*ECap*+_). In row 3 we have shape differences between *BCR* ^+^ and *BCR* ^−^ population when there is no extracapsular extensions (*BCR* ^−^_*ECap*−_ vs *BCR* ^+^_*ECap*−_). In each row the sagittal, the coronal, the axial and the posterior views are presented in that order.

In all three atlas comparison (*BCR* ^−^ vs *BCR* ^+^, *BCR* ^−^_*ECap*−_ vs *BCR* ^+^_*ECap*−_ and *BCR* ^−^_*ECap*+_ vs *BCR* ^+^_*ECap*+_) statistically significant differences in prostate capsule shapes were observed. The shape difference location however varied from one analysis to the other as observed in Fig. 3. Between *BCR* ^−^ and *BCR* ^+^ groups, statistically significant shape differences were identified on the anterior, posterior and the lateral side of the prostate as observed in 1st row of Fig. 3. Comparing patients with ECap (*BCR* ^−^_*ECap*+_ vs *BCR* ^+^_*ECap*+_) (2nd row of Fig. 3), significant differences were observed primarily towards the anterior side, but also towards the lateral and the posterior sides of the prostate. Comparing patients without ECap (*BCR* ^−^_*ECap*−_ vs *BCR* ^+^_*ECap*−_) (3rd row of Fig. 3), significant shape differences were identified towards the posterior side and also towards the lateral side. In all the experiments, shape differences towards the posterior of the prostate capsule were discernible along with differences towards the lateral side.

**Figure 4:**
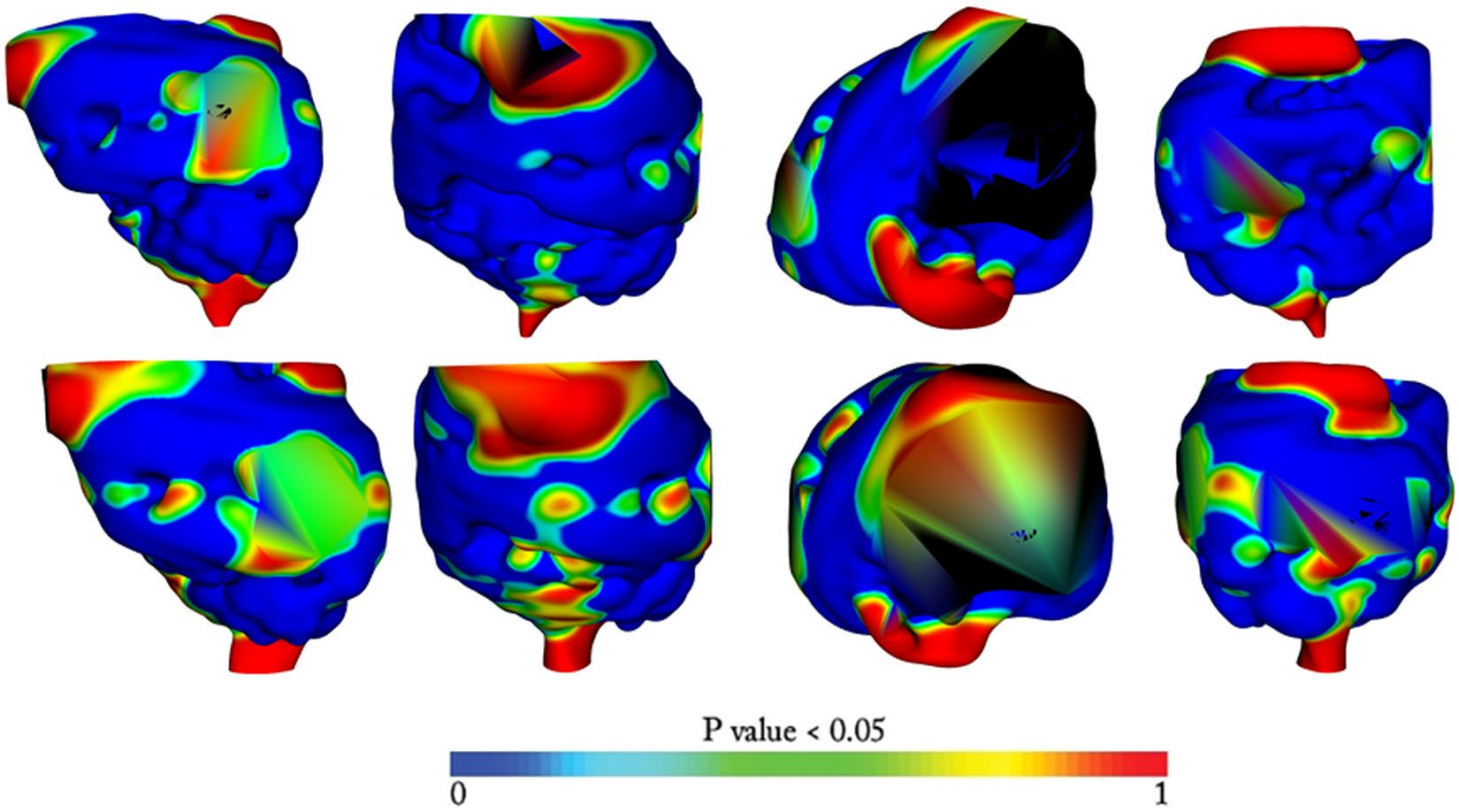
Shape differences of the prostate capsule with atlases created after registration of all patients to *BCR* ^−^ template (*A*) that was not present in the 25 patient *BCR* ^−^ cohort. All p-values are mapped to atlases and thresholded over 0.95. Blue value signifies low difference and red value signifies a bigger difference. The color bar is provided for reference. In row 1 we have shape differences when all *BCR* ^+^ and *BCR* ^−^ cases are compared. In row 2 we have shape difference between *BCR* ^−^ and *BCR* ^−^ with extra capsular extensions (*BCR* ^−^_*ECap*+_ vs *BCR* ^+^_*ECap*+_). In each row the sagittal, the coronal, the axial and the posterior views are presented in that order.

All *BCR* ^−^ and *BCR* ^+^ patients were also registered to *A* to analyze the sensitivity of the choice of template on the subsequent statistical analysis. Interestingly *BCR* ^−^ vs *BCR* ^+^ and *BCR* ^−^_*ECap*+_ vs *BCR* ^+^_*ECap*+_ exhibited similar statistically significant shape differences as observed in Fig. 4. No statistically significant shape differences were however observed between *BCR* ^−^_*ECap*−_ vs *BCR* ^+^_*ECap*−_. The results are presented in Fig. 4.

## Discussion

Aggressive cancers are known to induce a field effect that could affects large areas of cells on a tissue surface. This suggests that local deformation induced by the tumor as it grows could cause distensions in regions distant from the tumor, presumably even the surface of the organ within which the tumor is growing. BCR is often associated with aggressive cancer growth, one that might induce a field effect deformation^14,15^. In other words a tumor that is expanding and growing might be inducing stresses on the surrounding tissue which in turn might ripple over to the surface of the capsule. Thus a tumor field effect might manifest in the form of distensions to the capsule surface and additionally more and less aggressive tumors might differentially distend the capsule surface. On a T2w MRI scan, this distension might present as an irregular bulge and/or focal capsular retraction.

To the best of our knowledge, field effect that is strongly correlated to recurrence, has not been rigorously studied or investigated in the context of prostate cancer recurrence and prognosis. In this work, we focused on evaluating whether more and less aggressive prostate cancers (i.e. tumors that subsequently resulted in disease recurrence or not) could differentially induce changes and distensions to the surface of the prostate capsule.

Some works have shown the link between aggressive prostate cancers and extent and biochemical recurrence^7,8^. ECap extent are characterized by smooth and/or irregular capsular bulge and focal capsular retraction^8^. These shape features are often difficult to quantify and compare across population. Thus we adopt an atlas based statistical comparison approach to identify regions on the prostate that are affected and are different between *BCR* ^+^ and *BCR* ^−^ prostate cancer patients.

In our experiments a statistically significant shape differences between *BCR* ^+^ and *BCR* ^−^ patient atlases was observed. These differences hold when the atlases were created by registering to two separate templates or when registered to *A* (*BCR* ^−^ template outside the cohort). In both the experiments significant shape differences were observed in presence of extra-prostatic extent. The shape differences were not observed when all patients were registered to *A* and controlled for extra-prostatic extension. This may suggest that extra-prostatic extension is a possible contributor to the observed shape differences between the two cohorts.

The goal of the study was to evaluate and study shape differences of the prostate capsule as observed in atlases between *BCR* ^+^ and *BCR* ^−^ prostate cancer patients. Two groups based on Gleason score and tumor stage and equal number of patients were carefully selected to minimize the bias in statistical comparison. A long follow up time period of 5 years ensured that all *BCR* ^+^ and *BCR* ^−^ patients were correctly selected for the cohorts. No significant differences in prostate volume was observed between *BCR* ^+^ and *BCR* ^−^ cohorts.

A non-parametric GLM based t-test with multiple comparison was carried out to establish statistical significances of the voxels. Permutation testing and multiple comparison reduced the chance of false discovery error. Statistical comparison was dependent on the registration accuracy of the method adopted for building the *BCR* ^+^ and *BCR* ^−^ atlases. A high DSC and a low MASD values however suggest that the registration step is probably sufficiently accurate for the atlases used for statistical comparison.

Statistically significant differences in the shape of the prostate capsules were observed between *BCR* ^+^ and *BCR* ^−^ prostate cancer patients, mostly localized close to the posterior part of the prostate. Experiments were also conducted controlling for presence of ECap and to evaluate shape differences between the atlases of *BCR* ^−^_*ECap*−_ and *BCR* ^−^_*ECap*−_. Statistically significant shape differences were observed towards the posterior and the lateral side of the prostate, suggesting that there are capsule shape differences between *BCR* ^+^ and *BCR* ^−^ patients, independent of the presence or absence of extra-capsular spread. As observed in Fig. 3, the location of the significant differences in shape between the *BCR* ^+^ and *BCR* ^−^ atlases varied as a function of the patients comprising the atlases (see rows 1, 2 and 3). This is likely due to the presence or absence of extra-capsular extent within the patients used for creating the sub-population atlases.

The results varied with *BCR* ^+^ and *BCR* ^−^ cohorts registered to a common template *A* prior to atlas creation. Statistically significant differences were observed in atlas comparison between *BCR* ^−^ vs *BCR* ^+^ and *BCR* ^−^_*ECap*+_ vs *BCR* ^+^_*ECap*+_. This appears to further validate our initial finding on the presence of statistically significant shape differences between the *BCR* ^+^ and *BCR* ^−^ cohorts. No such statistical differences were observed when controlled for the ECap extension i.e. between *BCR* ^−^_*ECap*−_ vs *BCR* ^+^_*ECap*−_ atlases registered to a *BCR* ^−^ template. There could be two possible reasons for this; (1) The differences in prostate capsule shapes were primarily driven on account of extra-capsular spread. Thus controlling for ECap extension and registering all patients to *BCR* ^−^ template mitigates the influence of surface specific differences. (2) The surface differences that may have been present between the *BCR* ^−^_*ECap*−_ vs *BCR* ^+^_*ECap*−_ patients was diluted or lost due to registration of all the patients to the *BCR* ^−^ template.

Note that, though a high registration accuracy was observed, the registration framework may not account for local deformations of the prostate surface that may have been resulted from extra-prostatic extension. This may suggest that the detected differential distension observed between the BCR+ and BCR− cohorts may be sensitive to choice of the registration algorithm. For example a surface based thin-plate spline registration may dilute the distensions as observed in this study.

The results of the study hinged on the fidelity of the manual contouring of the capsules by the human readers. One of the concerns therefore was the reproducibility of the manual annotations of the capsule by a human reader in presence of different level of experiences and personal biases. We evaluated inter-observer variability of prostate capsule contouring by two human experts in terms of Dice similarity coefficient, (DSC), mean absolute surface distance (MASD) and center of gravity (COG). The DSC, MASD and COG were used for registration accuracy validation of the atlases as explained in Section 0.2.2. The DSC between the two experts was found to be 0.90 ± 0.02, MASD was found to be 0.75 ± 0.41 mm and COG difference was 2.13 ± 0.51 mm. Wilcoxon signed rank testing corrected with multiple comparisons did not reveal any statistically significant differences in the DSC, MASD and COG values between the expert contours. Thus we may assume that the subsequent shape differences observed between the *BCR* ^+^ and *BCR* ^−^ populations was not an artifact of a single reader’s annotation.

Our study did however have its limitations. In this preliminary study, a single center small cohort study we were unable to investigate the effect of change of magnet and acquisition parameters on contouring and hence the model. Prostate gland shape may be affected by both cancer and other natural causes like patient anatomy and other benign inflammations. Clearly, we will also need to evaluate the presence of these benign tumor confounding pathologies in future work.

In this preliminary study prostate capsule shape differences were observed between atlases of *BCR* ^+^ and *BCR* ^−^ patients. The study suggest that irregular prostate capsule surface may be used as a biomarker in addition to clinical variables like the PSA, Gleason score, T-stage to ascertain risk of BCR. In our future work shape features like curvature magnitude and surface normal orientations, along with radiomic features and clinical varibales will be used for BCR prediction with validation performed on a new holdout dataset.
